# Identifying and Taking Action on the Protective and Risk Factors of Black Maternal Mental Health: Protocol for Community-Based Participatory Study

**DOI:** 10.2196/70076

**Published:** 2025-06-16

**Authors:** Priscilla N Boakye, Kenneth Fung, Mawuko Setordzi, Egbe B Etowa, Rosanra Yoon, Josephine Etowa, Feven Desta, Nana Ama Tiwaa-Boateng, Modupe Tunde-Byass, Janet Yamada, Karline Wilson-Mitchell, Cynthia Maxwell, Crystal T Clark, Josephine Pui-Hing Wong

**Affiliations:** 1 Toronto Metropolitan University Toronto, ON Canada; 2 University of Toronto Toronto Western Hospital Toronto, ON Canada; 3 Western University London, ON Canada; 4 University of Ottawa Ottawa, ON Canada; 5 University of Toronto North York General Hospital Toronto Canada; 6 University of Toronto Women’s College Hospital Toronto, ON Canada

**Keywords:** Black mothers, Canada, community-based research, maternal mental health, mixed methods

## Abstract

**Background:**

Maternal mental health disorders are associated with adverse maternal and infant health outcomes. Despite advances in screening and treatment, disparities in maternal mental health disorders continue to disproportionately affect Black mothers and birthing persons. While there are studies that have examined maternal mental health, a gap in research remains in understanding the protective and risk factors of Black maternal mental health in Canada. Identifying the risks and protective factors is critical for advancing equitable and inclusive policies and practices that promote maternal well-being and optimal outcomes for Black perinatal populations.

**Objective:**

This paper presents an outline of a study protocol that seeks to identify the protective and risk factors of Black maternal mental health and to engage Black mothers and birthing persons from the Greater Toronto Area in codesigning a culturally safe and inclusive best practices model to inform policy and interventions.

**Methods:**

The proposed study will use an exploratory 3-phase sequential mixed methods approach underpinned by the principles of health equity and community-based participatory research. Phase 1 will involve engaging Black mothers and birth persons (n=300) in a survey to examine the psychosocial determinants of Black maternal mental health, including depression, anxiety, discrimination, strong Black women trope, attitude toward seeking mental health, support, and stigma. In phase 2, we will conduct 6 focus groups and individual interviews (n=60) to explore the stressors in the context of Black mothers and birth persons’ everyday lives, psychosocial and support needs, and conditions that promote their resilience. Finally, phase 3 will engage Black women and birthing persons (n=30) in a codesign session using the concept mapping method to identify priority areas for action to inform policy and programming. We will use SPSS version 26 (IBM Corp) to analyze the survey data, drawing on both descriptive and inferential statistics. NVivo (Lumivero), a qualitative data analysis software, will be used to organize the data from phase 2 into meaningful themes informed by Braun and Clarke’s thematic analysis approach.

**Results:**

Ethics approval was granted in July 2024. Data collection for phase 1 started in December 2024 and will be completed in April 2025. Findings from phase 1 will inform phases 2 and 3 of this study, which will be conducted in the third quarter of 2025. We will disseminate the results of this study in the second and third quarters of 2025.

**Conclusions:**

The findings will generate the much-needed knowledge to shift policy, practice, and research and support capacity building among Black mothers and birthing persons. In addition, the proposed study will contribute to informing policy initiatives and interventions at the health system and community level to advance mental health equity and build capacity among service providers to provide culturally safe and equitable mental health care.

**International Registered Report Identifier (IRRID):**

PRR1-10.2196/70076

## Introduction

### Background

Maternal mental health (MMH) disorders before and after birth are a major public health concern associated with poor pregnancy and mental health outcomes for parents [1-3]. Research also shows that poor MMH undermines the development of secure infant attachment, which in turn affects the development of socioemotional skills of children [4,5]. While all populations can be affected by poor MMH, research has consistently shown that Black mothers and birthing persons are at a higher risk of MMH disorders, including postpartum depression and anxiety before and after childbirth [6-8]. Despite advances in perinatal mental health care, racialized health disparities continue to exist within and across countries. Evidence from the United States suggests that 40% of Black mothers and birthing persons are more likely to experience MMH issues compared with White women [9]. In Canada, 23% of all women experience MMH disorders [1] with Black, Indigenous, and other racialized people accounting for 40% of all reported cases [10]. Despite the highly preventable and treatable nature of MMH disorders, only 15% of those who experience symptoms of depression and anxiety receive the required care [10]. MMH disorders cost the Canadian health care system an estimated CAN $6.7 billion (US $4.9 billion).

Few studies have explored MMH disorders among Black populations in Canada, but disparities in diagnosis, delivery of care, and their relationship to health outcomes for Black women have been well documented in the United States [11,12]. Black mothers and birthing persons are known to be at increased risk of MMH disorders before and after childbirth, and these have been associated with complex socioenvironmental factors, including anti-Black racism, discrimination, low socioeconomic status (SES), neighborhood disadvantage, inadequate social support systems, and stressful life circumstances [7,8,13-17]. Even after accounting for known risk factors such as a history of depression, anxiety, and substance use, Black mothers and birthing persons are still at an increased risk for MMH disorders [7,16]. They face unrelenting gendered racial stress, and this structural disadvantage coupled with emotional and hormonal changes increases their risk of postpartum mood and anxiety disorders [15].

MMH disorders can lead to adverse effects before and after childbirth [7,18]. High levels of stress and anxiety related to untreated MMH disorders can interfere with fetal development and increase the risk of adverse pregnancy outcomes including preterm birth, low birth weight, and risk of infant mortality in the first year of life [9,19]. Furthermore, MMH disorder affects the health and psychosocial functioning of Black mothers and birthing persons and undermines their capacity to care for themselves and their infants [20]. This in turn can interfere with a mother’s responsiveness to infant needs and ability to engage in infant caregiving [4,21-23], thereby affecting the child’s emotional and cognitive development potentially leading to social and behavioral issues [21,22].

Black mothers and birthing persons face significant barriers accessing mental health support. They are less likely to receive treatment due to challenges such as fear of stigma, involvement in child welfare services, and limited financial support [9,13,14,18]. Limited understanding of MMH disorders along with a lack of support among partners and family members can further isolate Black mothers and birthing persons and affect their ability to seek care. The stigma of mental illness and the expectation to demonstrate strength in the face of these negatively perceived issues can also create barriers for Black mothers to openly discuss their mental health and reinforce their hesitancy to seek care [11,12,24]. These factors are exacerbated by inadequate universal screening, low mental health literacy, lack of culturally relevant, gender-responsive care, systemic racial discrimination, microaggressions, and a fractured health system [7,11,12,24,25]. Untreated postpartum mental health disorders are known to increase the risk of maternal infanticide [26] and most recently have been identified as the leading cause of direct maternal death during pregnancy and within the first year of childbirth [27]. This is highly concerning given the already limited social support, lack of culturally responsive services, and overstrained community resources [9,11,12,28].

Research has shown that identifying and strengthening protective factors including coping resources and support systems can decrease the risk of postpartum depression among Black mothers and birthing persons [11]. While these findings from the United States provide valuable insights, they cannot be directly extrapolated to the Canadian context because the 2 countries differ fundamentally in terms of their social, structural, environmental, and health care systems. Some studies in Canada have explored perinatal mental health among women [29], but very few studies have specifically examined the protective and risk factors of MMH among Black mothers and birthing persons in the first year postpartum. In addition, there were significant gaps in Canadian literature on the cross-cultural considerations in response to and treatment of MMH disorders [29]. This limited evidence on Black MMH in Canada constitutes a significant barrier to promoting and advancing mental health equity. Given the significant public health concerns related to MMH disorders among Black mothers and birthing persons and the downstream impacts on infants before and after birth, it is crucial to identify and act on the risk and protective factors. Black mothers and birthing persons face complex intersectional issues and unique stressors that increase their risk of MMH disorders in the first year of postpartum [7,11,15]. Our project will contribute to increasing awareness and understanding of the protective and risk factors of MMH disorders for Black mothers and birthing persons and identify priority areas for action to advance MMH equity.

### Objectives

The overall goal of this study is to examine the protective and risk factors for MMH disorders among Black mothers and birthing persons and to critically engage them in identifying priority areas for action to advance MMH equity in Ontario and across Canada. The specific research objectives (RO) are:

- RO 1: Identify the protective and risk factors related to MMH disorders among Black mothers and birthing persons in the first year of postpartum
- RO 2: Explore the stressors in the context of everyday life, psychosocial and support needs, and conditions that promote resilience
- RO 3: Identify the factors that facilitate or impede MMH support
- RO 4: Engage Black women and birthing persons in identifying priority areas for action to inform policy and programming

### Theoretical Framework

This proposed study is underpinned by the principles of health equity as it relates to health outcomes of systematically marginalized and racialized communities. It is also informed by intersectionality [30] and socioecological frameworks [31]. Intersectionality is based on the understanding that (1) social identities constructed through power relations are not distinct and unidimensional but rather multiple and intersecting; (2) the experiences of historically oppressed and systematically marginalized groups are the focus of understanding; and (3) multiple social identities such as race, gender, and SES intersect and interlock with structural factors such as sexism, racism, and poverty to create disparities in health outcomes [30]. Intersectional analysis is based on the understanding that disparities in health outcomes do not occur in isolation but are a combination of complex interlocking conditions that contribute to inequality and inequity within society. Intersectional analysis enables researchers to analyze how racism, gender, economic marginalization, sexism, education, and neighborhood shape experiences and vulnerabilities associated with MMH disorders. In addition, we will draw on the socioecological framework to examine how individual, interpersonal, community, and societal conditions interact to influence MMH outcomes [31]. The socioecological framework situates the analysis of social and structural determinants of health across multiple levels to determine their effect on health and mental well-being and to identify areas for creating change [30]. Together, these frameworks will help the team identify the sociocultural, contextual, and structural conditions that shape the MMH of Black mothers and identify target areas for creative and sustainable intervention programs to strengthen protective factors and reduce risk factors to improve health outcomes.

## Methods

### Overview

**Figure 1:**
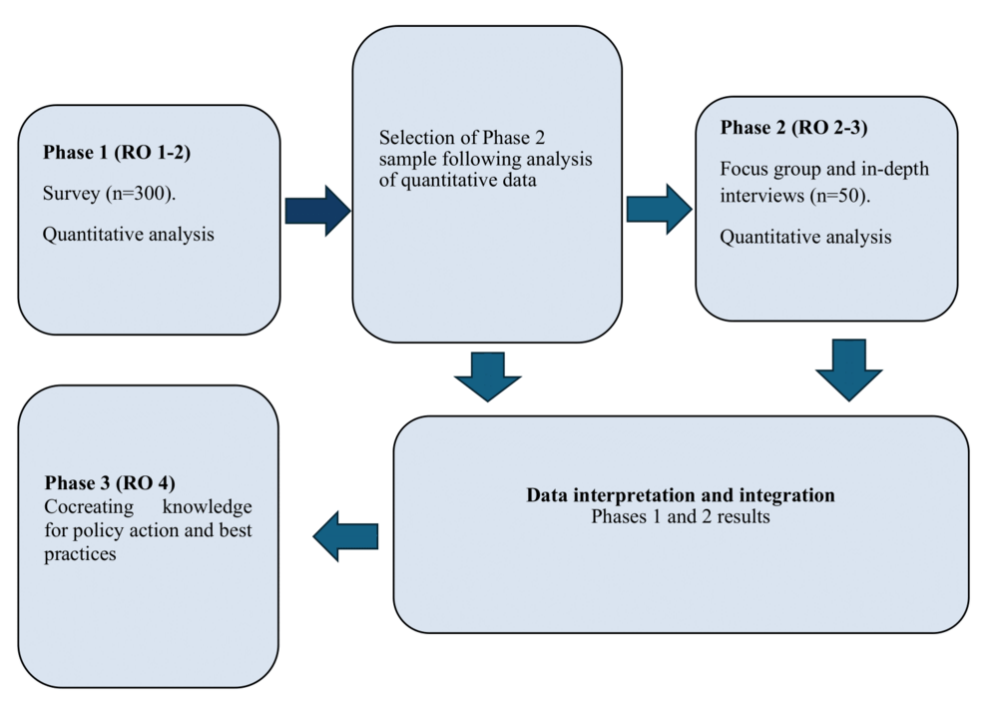
Sequential mixed methods approach.

The proposed study will use an exploratory 3-phase sequential mixed methods approach [32]. The approach outlined in Figure 1 will be used to identify and explore the protective and risk factors of Black MMH and to co-create best practices framework and policy action. The determinants of MMH disorders are complex and are shaped by broader social and political conditions at the individual, community, and societal levels. Therefore, understanding and taking action to reduce risks and strengthen protective factors requires a research approach that fosters collaboration and meaningful community engagement. The study will be guided by tenets of community-based participatory research. As an innovative and transformative research approach, community-based participatory research aims to generate understanding and action on issues affecting communities to reduce disparity and advance health equity [33]. Community-based participatory research draws on the principles of empowerment and meaningful engagement with communities, researchers, and interest holders in cocreating culturally transformative, sustainable, and equity-driven action to create change [34,35]. A combination of quantitative (sociodemographic survey and validated scales) and qualitative (focus group and in-depth interviews) methods and a participatory process of group concept mapping will be used to generate a new understanding of the complexities of Black MMH.

### Sample and Setting

We will foster meaningful community engagement by working with formal and informal collaborators in Black communities made up of Black mothers and birthing persons, community advocates, service providers, and faith leaders in Toronto, Canada. The inclusion criteria for participation are Black mothers and birthing persons who (1) self-identify as Black, (2) have biologically conceived and given birth within the past 12 months, (3) are caring for an infant 1 year or younger, and (4) speak English and reside in the Greater Toronto Area, Ontario. To enhance the efficiency of the data collection, we will purposively recruit 300 eligible Black mothers and birthing persons of all gender and sexual identities (self-identified as heterosexual, lesbian, bisexual, queer, cisgender, transgender, intersex, nonbinary, etc) and relationship and family compositions (same sex, intergenerational, sole parenting, etc). Recruitment will be supported by community partners across the study site and facilitated by the use of the study flyer in addition to using multiple approaches, including local public health agencies, diverse Black parenting groups, faith-based organizations, and a wider network of interest holders in the Black communities to promote participation and engagement (Black businesses, salons, Black churches, mosques, community, and neighborhood associations, etc). We will leverage social media platforms to ensure representation and will engage local public health agencies to disseminate information and recruitment flyers about this project.

### Phase 1: Objectives 1 and 2: Surveys on Mental Health Status and Coping

In this phase, an e-survey (n=300) will be used to collect data on the participants’ demographic (age, racial and ethnic identities, immigration status, educational status, SES, gender identity, housing, neighborhood, employment, relationship status, and household income along with obstetric information including gestational age at birth (full or preterm), admission to neonatal intensive care unit, and infant health issues or concerns. The following validated scales will be used to assess experiences of Everyday Discrimination Scale (EDS) [36], stress in the context of their everyday lives using the Parental Stress Scale (PSS) [37], mental health status using the 7-item Generalized Anxiety Disorder (GAD-7) [38] and Center for Epidemiological Studies-Depression (CES-D) [39], emotional support using Multidimensional Scale of Perceived Social Support (MSPSS) [40], attitude toward help-seeking mental health [41], and endorsement of the Strong Black Woman Archetype [42]. As an exploratory study with a Black population that is geographically spread out across the Greater Toronto Area, we will use convenience sampling techniques to reach our target population.

### Quantitative Data Analysis

Descriptive and inferential statistics will be used to analyze the data from the surveys. To identify risk and protective factors, stepwise linear regression analysis will be used to examine the impact of parental age, racial and ethnic identities, gender identities, educational status, SES, housing, neighborhood, employment, relationship status and family composition, discrimination, support, gestational age at birth on parental stress, anxiety, and depression. Intersectionality will be explored by examining interaction terms in the regression analysis, such as sexuality and SES, and race and SES.

### Phase 2: Objectives 2 and 3: Exploring the Experiences and Perspectives of Participants

In phase 2, we will conduct focus groups and in-depth individual interviews to explore the unique and common experiences of Black mothers and birthing persons (n=60) in the context of physical, emotional, psychological, social, and economic needs and overall well-being. We will engage 40-50 Black mothers and birthing persons in focus groups. We will determine the sample size based on the ontological and epistemological assumptions of qualitative research and purposive sampling [43]. We will use purposive matrix sampling [43], which allows us to reach the maximum diversity of participants specific to the study population and the intended study outcomes. We will conduct 5-6 focus groups (8-10 Black mothers and birthing persons per group) across the study site. These focus groups will provide opportunities for participants to share and exchange information, leading to the generation of deeper, comprehensive, and collective understanding. Participants will be drawn from phase 1, and we will also use a matrix to guide recruitment to enrich the diversity of all focus groups in terms of relationship status, family composition, gender identities, neighborhoods, and SES. We also plan to conduct 8-10 individual interviews with some participants to further explore their experiences, including sensitive topics that may be difficult to discuss in a group setting [44]. We expect that combining focus groups and interview methods will lead to a more comprehensive understanding through the triangulation of data collection methods, ultimately contributing to the trustworthiness of the findings [45].

### Qualitative Data Analysis

We will use thematic analysis [46] drawing on both inductive and deductive processes to analyze the data. The analytic process will be organized using NVivo. We will draw on intersectionality and socioecological frameworks to analyze the complex ways by which race, gender, class, and other factors intersect to affect the mental health, coping, and resilience of Black mothers and birthing persons. We will develop a codebook based on the research objectives and questions (deductive), as well as ideas and perspectives generated by the participants (inductive). A systematic and iterative process will include (1) familiarizing and sorting the data, (2) generating a coding template to guide the analysis, (3) assigning codes to the dataset, (4) categorizing similar phrases and statements into clusters, (5) identifying and defining themes and relationship between the themes, and (6) creating a narrative to describe the data interpretation [46]. The analytic process will be conducted in collaboration with the project team members to ensure broader and collective interpretation and understanding is achieved. The combined results of phases 1 and 2 along with relevant evidence from existing literature will be used to inform digital integrated knowledge translation (iKT) and the team will collectively work with participants to translate the findings into practical solutions relevant to the contexts of their experiences and community realities to co-create a best practices framework.

### Phase 3: Objective 3: Cocreating Knowledge for Policy Action and Best Practices

Phase 3 of this study is guided by the principles of meaningful community engagement and iKT [47]. In this phase, we will use concept mapping to engage Black mothers and birthing persons in cocreating an inclusive best practices model to inform policy and programming to promote mental well-being and resilience among Black mothers and birthing persons. We will use the Groupwise software (OpenText) to support the concept mapping activities. Concept mapping is an innovative community-based research method that has been used successfully to explore health and social issues from the perspective of affected individuals and communities [47,48]. Concept mapping has been used to understand and develop equity-driven best practice guidelines and models to improve the health of communities [49]. As a strategy for iKT and knowledge cocreation, concept mapping will facilitate the uptake of evidence generated in phases 1 and 2 and engage Black mothers and birthing persons to collectively translate the evidence into actionable strategies.

### Sampling

We will use purposive matrix sampling to recruit diverse Black mothers and birthing persons (n=30). According to the researchers who developed the Group Concept Mapping methods and software, 20-30 participants are adequate for effective use of concept mapping [49]. We will draw from the lists of phase 1 and 2 participants who have indicated their wish to participate in phase 3. In addition, we will engage Black mothers and birthing persons who are interested but not able to take part in phases 1 or 2. Using the matrix methods, we will recruit a group of diverse Black mothers and birthing persons based on their relationship status, family composition, ethnic ancestry, gender identities, neighborhoods, and SES.

### Concept Mapping: Data Collection, Map Analysis, and Interpretation

As a structured process, concept mapping captures participants’ responses and ideas on a given issue and translates them into an interpretable visual representation (concept map). All ideas are displayed through the concept map, which shows the relationship between the ideas and the degree of significance and importance. When engaging participants in developing the best practices framework, we will apply intersectionality as a guiding framework to integrate gender- and sex-based analysis with race and class or SES-based analysis. The concept mapping process with Black mothers and birthing persons will involve 4 meetings via Zoom, and we will use the Groupwise software and group discussion to support data collection, analysis, and interpretation. The concept mapping process allows for consensus building and collaboration with participants:

### Session 1: Knowledge Exchange

The combined results of phases 1 and 2 along with relevant evidence from existing literature will be presented by the research team, and participants will be invited to respond to these findings in relation to their lived experiences. Both research teams and participants will engage in dialogue to further explicate the social determinants and intersectional experiences of Black mothers and birthing persons. This dialogue will prepare participants to co-create a best practices framework in subsequent sessions.

### Session 2: Brainstorming

Drawing on the study results presented in session 1, participants will engage in brainstorming ideas for best practices to meet the needs of Black mothers and birthing persons. They will be given a prompt, “What needs to be done by various stakeholders (individuals, families, service providers, community organizations, and government and policymakers) to promote the MMH of Black mothers and birthing persons,” and work individually to generate as many ideas as they can based on the prompt. We anticipate that participants will generate statements of ideas on effective and culturally safe programs and services, community support and resources, health service delivery, policy actions, and socioenvironmental conditions that promote or hinder resilience. The research team will refine the statements generated to remove redundant statements, and these will be entered into the Group Concept Mapping computer program to be aggregated based on similarities.

### Session 3: Sorting and Rating

In this session, each member will be given a list of statements generated by the Group Concept Mapping program. They will work individually to sort the itemized ideas into stacks according to similarity and provide each stack with a name. Participants will also be given a list of items to rate based on importance using a scale of 1-5, with 5 being the most important. Responses from participants’ input will be entered into the Group Concept Mapping program to generate concept maps for interpretation.

### Session 4: Analysis and Interpretation of Concept Maps

The analysis includes (1) multidimensional scaling to generate point maps; (2) hierarchical cluster analysis to generate 3-dimensional cluster maps, individual items (statements) are collated into thematic maps to show the relative importance of each theme; (3) pattern matching analysis is used to compare importance and feasibility ratings for each cluster of strategies (statements); and (4) go zone plots and analyses will be used to show the graphical representation of the mean values obtained for all the individual statements in order of importance and feasibility rating scales. We will facilitate discussion with participants, assess the degree of alignment of the concept maps with their experience, and work collaboratively to make sense of these maps. After the initial large group interpretation of the clusters, participants will work in small groups of 4-6 to translate the maps into practical solutions relevant to the contexts of their experiences and community realities. They will then return to the large group, share their ideas, and work collectively to integrate all group ideas into a best practices framework. After completing the 4 sessions of group concept mapping, the research team will finalize the best practices framework and translate it into audience-specific products to promote effective dissemination and uptake.

### Team Expertise

Our team consists of a multidisciplinary team of researchers with outstanding track records in community-based action research in the areas of mental health and health equity. The team comprises senior and early career researchers in academic institutions and clinical and community-based settings, many of whom have experience working with Black women in the prenatal and postnatal periods in addition to scholarship on anti-Black racism and health inequities experienced by Black communities. Collectively, the team brings diverse expertise and experience including quantitative research, qualitative, and mixed methodologies; cultural psychiatry, and sociocultural issues in mental health; community-based action research and knowledge translation and exchange; and implementation science. The research team also includes community partners and collaborators with grassroots connections with our population of interest, who will support the successful implementation of this project.

### Ethical Considerations

The protocol for this study has been reviewed and approved by the Toronto Metropolitan University Research Ethics Board (REB 2024-126). Data will be collected over a period of 1 year starting December 2024 to May 2025. The team will collectively adhere to ethical principles and guidelines governing the study protocol during phases of the project implementation including informed consent, privacy and confidentiality, and rights of participants. Participants will be asked to provide written informed consent before completing the survey, focus groups, in-depth interviews, and concept mapping sessions. Data from the focus groups and in-depth interviews will be purged of any identifiable information before being stored in an institutional repository. Each participant will receive CAN $30 (US $22) for completing the survey, and those who will be invited to participate in the focus groups and interview will receive CAN $50 (US $36).

## Results

This study received ethics approval from a Canadian institution in July 2024, and data collection for phase 1 started was December 2024 and was completed at the end of April 2025. A total of 329 participants completed the e-survey. The data is currently being processed for analysis using SPSS version 26, and the findings will be used to inform the development of the focus group guide for phase 2 and the co-creation process for phase 3. We anticipate the results of this study will be available in the third and fourth quarters of 2025.

## Discussion

### Overview

This research seeks to examine the protective and risk factors for Black MMH in the Canadian context. The new evidence will contribute to addressing the long-standing research gaps and inform change in policy, research, and practice to advance health equity. While research on risk factors of MMH among Black women is well documented [24], very few studies have identified factors that promote resilience and protect against perinatal mood disorders [50,51]. Moreover, maternal mental disorders are shaped by complex interactions of social and structural determinants. This proposed project will be among the first few studies to identify the risk and protective factors of MMH among Black mothers and birthing persons in Canada. Reducing the risk associated with MMH disorders and strengthening protective factors are critical to addressing the disparities in mental health outcomes that disproportionately impact Black women during pregnancy and after childbirth. Based on this understanding, we will use a mixed methods study design (quantitative and qualitative methods) informed by the principles of a community-based participatory research approach to engage Black mothers and birthing persons, interest holders, community leaders, service providers, decision makers, policy and mental health advocates, and parent champions to co-create best practices framework to reduce MMH disparities and advance equity. The involvement of different stakeholders contributes to narrowing the knowledge gaps on the experiences and mental health needs of Canadian Black mothers and birthing persons and offers evidence to advance inclusion. We will discuss the findings of this study in the context of current evidence, highlighting the similarities and contrasting perspectives.

### Dissemination of Findings

The findings of this study will be disseminated widely through conferences and community networks. We will disseminate the evidence to researchers through peer-reviewed publications and presentations at local, national, and international conferences. The findings will also be translated into policy briefs, infographics, and e-posters to be disseminated to policy makers, share knowledge with the public through media interviews (eg, CBC Metro Morning, Black community) and social media to communicate to wider stakeholders to stimulate policy priorities and change. We will also present our findings to Black community groups and organizations to identify needs and priorities for meaningful action.

### Strengths and Limitations

The proposed study has strengths. First, the integration of quantitative and qualitative methods will contribute to a comprehensive understanding of the protective and risk of Black MMH. In addition, using a mixed methods approach provides multiple and contextual insights that will enhance the knowledge cocreation and identify priority areas for action and programming. Potential limitations include those that can be complex and challenging to reconcile. We also anticipate that recruitment may be challenging given that the Black mothers and birthing persons are geographically dispersed throughout our research study setting. However, we have established a network of collaboration with community partners and collaborating organizations to facilitate recruitment.

### Conclusion

This project is well positioned to shift the deficit approach to understanding Black MMH, generate the much-needed critical knowledge, and increase awareness of the protective factors of MMH. Using an innovative concept mapping approach as an engagement and research tool, this study will contribute to the development of an effective policy and culturally relevant best practices model, and guide the design of interventions to optimize and promote equitable access to mental health support for Black mothers in Canada and beyond.

## Data Availability

The data to be generated by the study will be encrypted and stored securely in an institutional repository located at the Toronto Metropolitan University for a period of 5 years and will be available to other researchers upon reasonable request.

## Appendix

Multimedia Appendix 1

## Abbreviations

CES-D: Center for Epidemiological Studies-Depression
EDS: Everyday Discrimination Scale
GAD-7: 7-item Generalized Anxiety Disorder
iKT: integrated knowledge translation
MMH: maternal mental health
MSPSS: Multidimensional Scale of Perceived Social Support
PSS: Parental Stress Scale
SES: socioeconomic status
