# Supplementary material for: Identifying and Taking Action on the Protective and Risk Factors of Black Maternal Mental Health: Protocol for Community-Based Participatory Study
Source: JMIR Res Protoc. 2025 Jun 16;14:e70076. doi: 10.2196/70076 (PMC12209720; doi:10.2196/70076)
Supplement: Multimedia Appendix 1 [file resprot_v14i1e70076_app1.pdf]

Review Type/Type d'évaluation: SO Notes /Notes de l'agent scientifique
Name of Applicant/Nom du chercheur: Boakye, Priscilla Nailatu
Application No./Numéro de demande: 508492
Agency/Agence: CIHR/IRSC
Competition/Concours: 2023-09-14 Project Grant/Subvention Projet
Committee/Comité: Psychosocial, Sociocultural & Behavioural Determinants of Health/Déterminants psychosociaux, socioculturels et comportementaux de la santé
Title/Titre: Understanding and taking action on the risk and protective factors of maternal mental health of Black Mothers/Gestational Parents

---

Assessment/Évaluation:

Strengths (including SGBA considerations):

Aims of improving mental health and promote resilience in this population was seen as a novel and needed area of work, where little research has been done.

Intersectionality and socioecological framework used to guide this research increased confidence in the likelihood these methods will result in translatable and transferable outcomes that will advance the field.

Co-creation project was appreciated: the concept mapping activity and follow-up multisectoral approach with community partners was thought to be a feasible and meaningful manner to engage and a priori plan their KT.

Research team holds ideal connections with community networks and well suited to conduct this research.

Methodological details that thoughtfully considered equity seen throughout the proposal was applauded.

Implications of this work to further future research in this area was seen as probable in part due to the KT activities embedded.

Past reviewer comments adequately addressed.

Weaknesses (including SGBA considerations):

Ambiguity and ethical concerns were raised regarding targeted sample: where is the sample size calculation and justification for all phases of research (quant, qual, and co-creation project), and is this ethically required for this many community member’s time, given the challenges that mothers/birthing parents already face?

Choice of stepwise regression analyses was questioned. The team is encouraged to explore more sophisticated statistics to address research questions.

Sex and gender considerations were not adequately discussed in the quantitative plan. The applicants are encouraged to consider whether it is possible to include gender identity in both quantitative and qualitative analyses. The proposal suggests intersectionality analyses, which they may not adequately powered.

Plan to support individuals who are experiencing high distress and depressive symptomatology in this

**Review Type/Type d'évaluation:**	SO Notes /Notes de l'agent scientifique
**Name of Applicant/Nom du chercheur:**	Boakye, Priscilla Nailatu
**Application No./Numéro de demande:**	508492
**Agency/Agence:**	CIHR/IRSC
**Competition/Concours:**	2023-09-14 Project Grant/Subvention Projet
**Committee/Comité:**	Psychosocial, Sociocultural & Behavioural Determinants of Health/Déterminants psychosociaux, socioculturels et comportementaux de la santé
**Title/Titre:**	Understanding and taking action on the risk and protective factors of maternal mental health of Black Mothers/Gestational Parents

Assessment/Évaluation:

population is encouraged.

Budget:

Trainee and staff support seen as excessive: cutting 1 Master’s student recommended. Applicants are encouraged to further justify their budget requests.
